# Fabrication of ZnO@Ag_3_PO_4_ Core-Shell Nanocomposite Arrays as Photoanodes and Their Photoelectric Properties

**DOI:** 10.3390/nano9091254

**Published:** 2019-09-03

**Authors:** Zao Yi, Xin Li, Hui Wu, Xifang Chen, Hua Yang, Yongjian Tang, Yougen Yi, Junqiao Wang, Pinghui Wu

**Affiliations:** 1 Joint Laboratory for Extreme Conditions Matter Properties, Southwest University of Science and Technology, Mianyang 621010, China (Z.Y.) (X.L.) (H.W.) (X.C.) (Y.T.); 2 State Key Laboratory of Advanced Processing and Recycling of Non-ferrous Metals, Lanzhou University of Technology, Lanzhou 730050, China; 3 College of Physics and Electronics, Central South University, Changsha 410083, China; 4 School of Physics and Engineering and Key Laboratory of Materials Physics of Ministry of Education of China, Zhengzhou University, Zhengzhou 450001, China; 5 Research Center for Photonic Technology, Key Laboratory of Information Functional Material for Fujian Higher Education, Quanzhou Normal University, Fujian 362000, China

**Keywords:** ZnO@Ag_3_PO_4_ nano-heterostructures, super hydrophilicity, photoelectric performance

## Abstract

In this study, we combine the methods of magnetron sputtering, hydrothermal growth, and stepwise deposition to prepare novel ZnO@Ag_3_PO_4_ core-shell nanocomposite arrays structure. Through scanning electron microscope (SEM) topography test, energy dispersive spectrometer (EDS) element test and X-ray diffractometry (XRD) component test, we characterize the morphology, element distribution and structural characteristics of ZnO@Ag_3_PO_4_ core-shell nanocomposite arrays structure. At the same time, we test the samples for light reflectance, hydrophilicity and photoelectric performance. We find that after deposition of Ag_3_PO_4_ on ZnO nanorods, light reflectance decreases. As the time of depositions increases, light reflectance gradually decreases. After the deposition of Ag_3_PO_4_, the surface of the sample shows super hydrophilicity, which is beneficial for the photoelectric performance test. Through the optical transient response test, we find that the photo-generated current reaches a maximum when a small amount of Ag_3_PO_4_ is deposited. As the time of depositions of Ag_3_PO_4_ increases, the photogenerated current gradually decreases. Finally, we conducted an alternating current (AC) impedance test and also verified the correctness of the photocurrent test. Therefore, the structure is expected to be prepared into a photoanode for use in fields such as solar cells.

## 1. Introduction

ZnO is a new type of semiconductor material with a band gap of 3.37 eV and an exciton binding energy of 60 mV [1]. Due to its excellent thermal conductivity, chemical stability and strong UV absorption capacity, ZnO nanocomposite structure has great applications potential in sensors, detectors, solar cells [2,3,4,5,6,7,8,9,10,11,12,13,14]. In recent years, due to the unique physical properties of low-dimensional ZnO, it has attracted widespread attention, especially in the field of photoelectric conversion. For example, ZnO one-dimensional nanorod arrays have superior optical properties and electron transport properties than ZnO thin films [15,16,17]. When ZnO nanomaterials are combined with other semiconductor materials, the formed heterojunction can effectively improve electron-hole pair separation ability and utilization of visible light, which is very important for the development of optical devices. Low-dimensional ZnO nanomaterials have a wide application in degradation of organic pollutants [18,19,20] due to their large specific surface area and more reactive sites. At present, methods for preparing ZnO nanomaterials include the hydrothermal method [21,22,23,24,25], the magnetron sputtering method [26,27,28,29], the chemical vapor deposition method [30,31,32,33], and electrochemical deposition [34,35,36]. In the process of actually preparing ZnO nanomaterials, the hydrothermal method stands out in various methods and has been widely used due to its advantages of no pollution and low cost.

Low-dimension nanomaterials like noble metal nanoparticles (NPs), carbon nanomaterials, and Ag_3_PO_4_ nanoparticles manifest many attractive properties, and they offer great potential applications for bioimaging, energy conversion, optoelectronic devices, wave absorption, and sensors [37,38,39,40,41,42,43,44,45,46,47,48,49,50]. Among them, as a new type of indirect bandgap n-type semiconductor, Ag_3_PO_4_ has a forbidden band width of 2.45 eV and is capable of absorbing ultraviolet and visible light and its quantum yield is over 90%. The edge potentials of the Ag_3_PO_4_ conduction band (CB) and the valence band (VB) are 0.4 eV and 2.9 eV, respectively, and their VB potential is 2.6 eV lower than ZnO [51]. The extremely low valence band potential gives the photogenerated holes a strong oxidizing power. Due to the band gap and band potential of Ag_3_PO_4_, it exhibits excellent photooxidation capability, including degradation of organic dyes under visible light and the production of oxygen [52,53]. So far, Ag_3_PO_4_ crystal materials have been prepared in a variety of morphologies, such as diamond dodecahedrons, cubes and spheres. The method for preparing Ag_3_PO_4_ includes the following: Coprecipitation method [54], chemical bath deposition method [55], and the hydrothermal method [56]. However, the optoelectronic properties of pure Ag_3_PO_4_ may still be limited by its photo-generated charge separation/transport efficiency and limited light trapping capability. According to previous reports, the target semiconductor is combined with an appropriate metal [57] or semiconductor [58,59] to form a specific composite heterostructure, which can effectively control the energy band structure and surface charge distribution of the composite heterostructure, thereby improving the overall photoelectric performance of the material.

In this study, we prepare ZnO@Ag_3_PO_4_ core-shell nanocomposite arrays structure by way of the hydrothermal method and the stepwise deposition method. Firstly, the ZnO seed layer is prepared by magnetron sputtering on conductive glass (FTO), then ZnO nanorods are prepared by the hydrothermal growth method. Finally, Ag_3_PO_4_ is deposited on the surface of ZnO nanorods by the stepwise deposition method. The deposition of Ag_3_PO_4_ on the surface of ZnO nanorods changes the hydrophilicity of the surface. Compared with a single ZnO nanomaterial, we simply prepared the ZnO@Ag_3_PO_4_ nanocomposite structure by hydrothermal method and stepwise dipping method. The formed nanocomposite structure can more reduce the light reflection and enhance the separation of electron-hole pairs, which has practical significance for the production of photovoltaic devices. When an applied external bias is applied to the photoanode of ZnO@Ag_3_PO_4_, the photogenerated electron-hole pairs are separated and then is taken out to the external circuit by external bias. The deposition of a small amount of Ag_3_PO_4_ can maximize the photo-generated current, so the structure can be applied to photoelectric switches, photodetectors and other fields.

## 2. Experiment

### 2.1. Materials

Conductive glass (FTO); deionized water; ZnO target (purity 99.99%, the diameter of 60 mm, from Zhongnuo New Material (Beijing) Technology Co., Ltd, Beijing, China); hexamethylenetetramine (HMTA, from Tianjin Zhiyuan Chemical Reagent Co., Ltd, Tianjin, China); acetone (from Tianjin Zhiyuan Chemical Reagent Co., Ltd, Tianjin, China); zinc nitrate hexahydrate (Zn(NO_3_)_2_·6H_2_O, from Shanghai Titan Chemical Co., Ltd, Shanghai, China); silver nitrate (AgNO_3_, from Shanghai Sansi Reagent Co., Ltd, Shanghai, China); disodium phosphate (Na_2_HPO_4_, from Chengdu Cologne Chemical Co., Ltd, Chengdu, China); absolute ethanol(from Chengdu Kelon Chemical Reagent Factory, Chengdu, China); Na_2_SO_4_ solution (0.1 mol/L).

### 2.2. Cleaning the Conductive Glass (FTO) Substrate

The FTO conductive glass was first ultrasonically cleaned with absolute ethanol for 10 min, and then ultrasonically cleaned with acetone for 10 min. Finally, the FTO conductive glass was ultrasonically cleaned with deionized water for 10 min and then dried.

### 2.3. Preparation of ZnO Seed Layer

In order to successfully grow ZnO nanorods on the surface of FTO, we first use magnetron sputtering to deposit a layer of ZnO seed on the FTO surface. The experiment was carried out under the conditions of a pressure of 4 × 10^−4^ Pa, the argon gas flow rate of 40 sscm, then a radio frequency sputtering power of 60 W. We first pre-sputtered for 5 min and then officially sputtered each sample for 2 min. After the sputtering experiment was completed, the samples were taken out after cooling for 60 min.

### 2.4. Preparation of ZnO Nanorods Array

We used the hydrothermal method to grow ZnO nanorods on FTO with a deposited ZnO seed layer. In the experiment, we mixed hexamethyltetramine and zinc nitrate hexahydrate in a ratio of 1:1 as a precursor solution. The concentration of the solution was 40 mmol/L, the reaction temperature was 95 °C, and the reaction time was 4 h. After the reaction time was completed, we took the sample and washed it with deionized water for 3 times, then allowed it to dry naturally.

The reaction in the solution system was supposed to be as follows:

(1) $${\left(CH_{2}\right)}_{6}N_{4}+H_{2}O\to HCHO+NH_{3}\cdot H_{2}O$$

(2) $$NH_{3}\cdot H_{2}O\to NH^{4+}+OH^{-}$$

(3) $$Zn{\left(NO_{3}\right)}_{2}+2NH_{4}OH\to Zn{\left(OH\right)}_{2}↓+2NH_{4}NO_{3}$$

(4) $$Zn{\left(OH\right)}_{2}+4NH_{4}OH\to Zn{\left(NH_{3}\right)}_{4}{}^{2+}+2OH^{-}+4H_{2}O$$

(5) $$Zn{\left(NH_{3}\right)}_{4}{}^{2+}+OH^{-}\to ZnO↓+4NH_{3}+H_{2}O$$

### 2.5. Preparation of Ag_3_PO_4_ by Stepwise Dipping Method

In the experiment, we used the stepwise deposition method to prepare Ag_3_PO_4_ material. We used AgNO_3_ and Na_2_HPO_4_ solutions as deposition solutions. The concentration and volume of the deposition solution were 0.02 mol/L and 200 mL, respectively. During each deposition process, the deposited sample was placed in the AgNO_3_ solution for 30 min, then transferred to the Na_2_HPO_4_ solution for 5 min, and finally rinsed 3 times with deionized water. After the experiment was completed, all samples were taken out and naturally dried in a fume hood for use.

### 2.6. Annealing Experiment

The sample was placed in an annealing furnace and heated from room temperature at a rate of 4 degrees Celsius per minute. And after the temperature was raised to 140 degrees Celsius, maintained the temperature for 80 min. The next step was to stop the program, then waited for it to slowly drop to room temperature automatically, and last took out the sample.

### 2.7. Characterization

The surface structure and morphological characteristics of the composite samples were characterized using a scanning electron microscope (ULTRA 55, Zeiss, Heidenheim, Germany). The samples were analyzed by X-ray diffractometry (D/max-1400, Japanese Science, Tokyo Metropolis, Japan). The light-reflecting properties of the samples were characterized by a solid ultraviolet-visible-near-infrared spectrophotometer (UV-3150, Shimadzu, Shanghai, China). The test wavelength range was 185–3300 nm with a resolution of 0.1 nm. The hydrophilic and hydrophobic properties of the samples were analyzed by a contact angle tester (DSA30, Kruss, Hamburg, Germany). The test contact angle ranged from 0 to 180° with a resolution of ± 0.01°.

### 2.8. Optical Performance Test

In the experiment, we used a sample with an effective area of 1 cm^2^ on the working electrode. We used 0.1 mol/L Na_2_SO_4_ solution (pH = 7) as the electrolyte, the electrode with 0.5 mm diameter platinum wire and the reference electrode with saturated calomel electrode (SCE). Finally we let the light source enter from the back of the sample. When testing the photo-generated current, we set the bias voltage to 0.3 V, the sampling time to 400 s, and the sampling interval to 40 s. When testing the alternating current impedance, we set the bias voltage to 0 and the frequency range was 10^−2^–10^5^ Hz.

## 3. Results and Discussions

The hexamethylenetetramine in the solution is first hydrolyzed to form NH_3_.H_2_O. OH^−^ and Zn^2+^ form precipitated Zn(OH)_2_, the precipitate dissolves under the action of OH^−^, and finally forms ZnO through the action of OH^−^ [15,16,17]. The formed ZnO finally forms ZnO nanorods along the ZnO seed layer on the FTO substrate. In the process of depositing Ag_3_PO_4_, the sample is first placed in AgNO_3_ solution to fully absorb Ag^+^ on the surface of the sample. In the Na_2_HPO_4_ solution, Ag^+^ and PO_4_^3−^ ions are combined to form Ag_3_PO_4_ precipitate deposit on the surface of ZnO nanorods.

**Figure 1:**
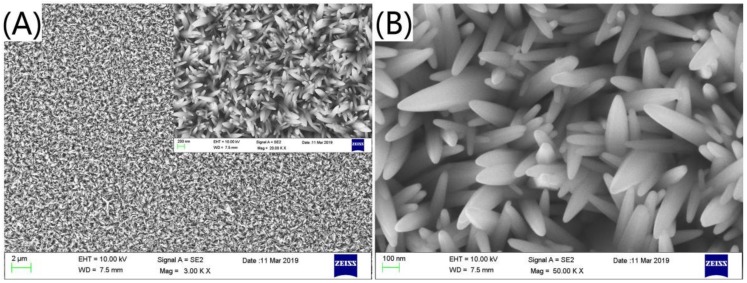
SEM image of ZnO nanorods at low magnification (**A**) and high magnification (**B**) viewing angles.

**Figure 2:**
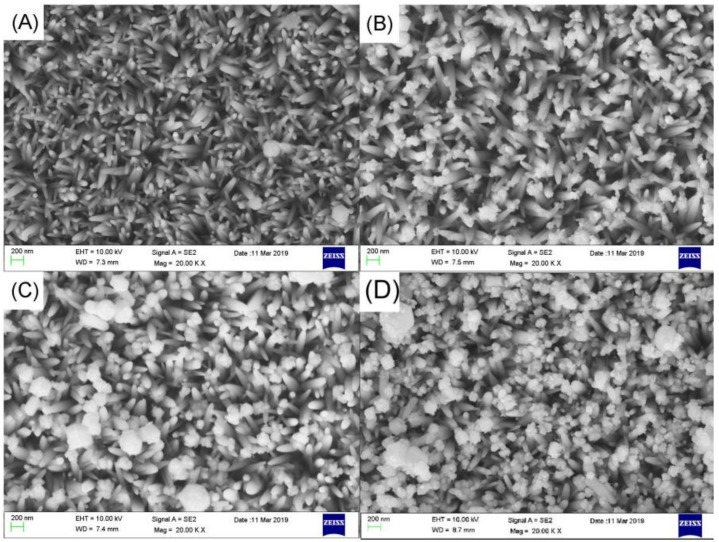
SEM images of ZnO nanorods+Ag_3_PO_4_: (**A**) Ag_3_PO_4_ deposition once, (**B**) Ag_3_PO_4_ deposition three times, (**C**) Ag_3_PO_4_ deposition five times, (**D**) Ag_3_PO_4_ deposition seven times.

In Figure 1, (A,B) are SEM images of FTO + ZnO nanorods. We can observe the large-area growth of ZnO nanorods and there is no connection between the nanorods. Figure 2 SEM images are FTO+ ZnO nanorods + Ag_3_PO_4_ (1 deposition, 3 depositions, 5 depositions, 7 depositions), corresponding to (A–D), respectively. It can be seen that under the condition of 1 deposition, a small amount of Ag_3_PO_4_ adhere to the surface of the nanorods from the Figure 2. As the increase of deposition times, it is observed that the amount of the Ag_3_PO_4_ is large and it grows into noticeable blocks. When the deposition time is 7 times, the amount of Ag_3_PO_4_ covers the nanorods almost completely forming large aggregates.

**Figure 3:**
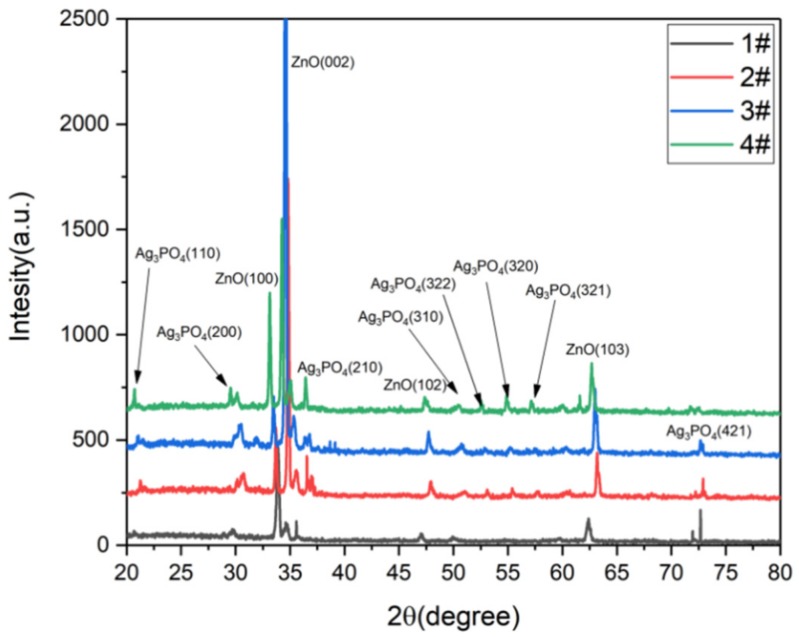
XRD pattern of ZnO nanorods+Ag_3_PO_4_: (1#) Ag_3_PO_4_ deposition once. (2#) Ag_3_PO_4_ deposition three times. (3#) Ag_3_PO_4_ deposition five times. (4#) Ag_3_PO_4_ deposition seven times.

In Figure 3, 1#, 2#, 3#, 4# correspond to FTO + ZnO nanorods + Ag_3_PO_4_ (1 deposition, 3 depositions, 5 depositions, 7 depositions). Under the hydrothermal method used the ZnO nanorods begin to grow on the seed substrate along the C axis. After deposition of Ag_3_PO_4_ on the surface of ZnO nanorods, lattice shrinkage is caused. The more the amount of deposited Ag_3_PO_4_, the more obvious the lattice shrinkage causes the diffraction peak to shift to a high angle. The diffraction peaks appearing in ZnO are present at diffraction angles of 31.75°, 34.39°, 47.50°, 62.80°, corresponding to its crystal plane (100), (002), (102), (103). The peak of the (002) crystal orientation is the strongest, indicating that ZnO grows preferentially along the (002) crystal plane. The Ag_3_PO_4_ characteristic peaks correspond to (110), (200), (210), (310), (322), (320), (321), (421), respectively [60,61]. As the increase of deposition times, the characteristic diffraction peak of the Ag_3_PO_4_ gradually increases. By comparing each diffraction peak of the Ag_3_PO_4_, it can be seen that Ag_3_PO_4_ grows uniformly in all directions.

**Figure 4:**
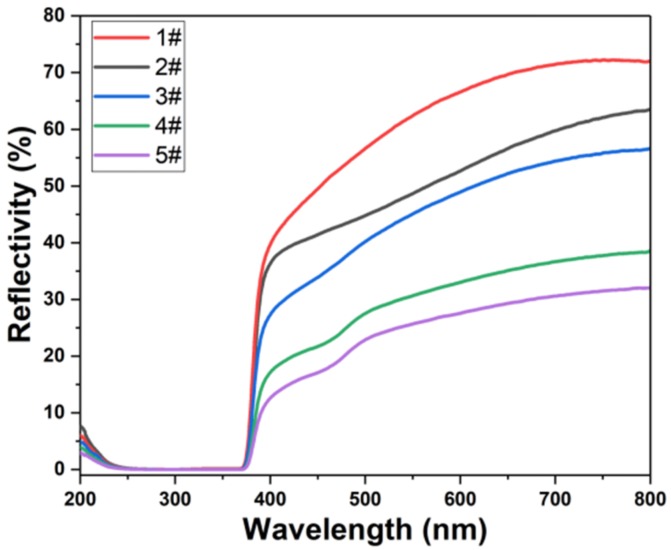
UV-visible reflectance of the sample: (1#) ZnO nanorods. (2#) ZnO nanorods + Ag_3_PO_4_ (1 deposition). (3#) ZnO nanorods + Ag_3_PO_4_ (3 depositions). (4#) ZnO nanorods + Ag_3_PO_4_ (5 depositions). (5#) ZnO nanorods + Ag_3_PO_4_ (7 depositions).

Figure 4 shows the UV-visible reflectance spectrum of the sample. We observed a higher reflectivity of ZnO nanorods in the visible range. After deposition of Ag_3_PO_4_, the reflectance in the visible light band gradually decreases. Since ZnO nanorods have strong light absorption in the ultraviolet light band [62], we can see from Figure 4 that the light reflectance in the 220 nm–380 nm band is almost 0. From Figure 4, we can observe that as the increase of deposition times of the Ag_3_PO_4_, the light reflection gradually decreases. When Ag_3_PO_4_ is deposited 7 times, the light reflection of ZnO nanorods is minimized. Since Ag_3_PO_4_ can effectively capture visible light and ultraviolet light of less than 530 nm, the light reflectance decreases rapidly around the 500 nm optical band. In general, the deposition of Ag_3_PO_4_ on the surface of ZnO nanorods can effectively reduce the reflection of light.

**Figure 5:**
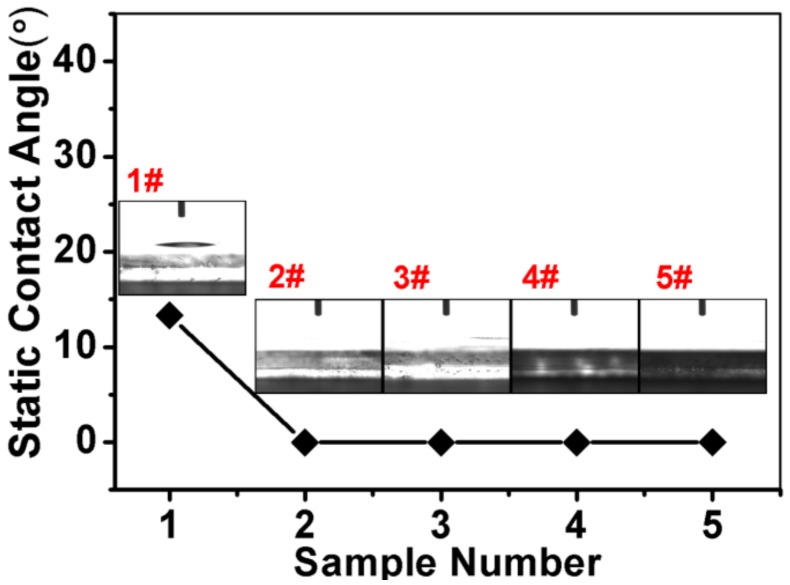
Static contact angle of the sample: (1#) ZnO nanorods. (2#) ZnO nanorods + Ag_3_PO_4_ (1 deposition). (3#) ZnO nanorods + Ag_3_PO_4_ (3 depositions). (4#) ZnO nanorods + Ag_3_PO_4_ (5 depositions). (5#) ZnO nanorods + Ag_3_PO_4_ (7 depositions).

Figure 5 shows the hydrophilicity of the sample. We observe that when only ZnO nanorods are grown on FTO conductive glass, the contact angle is about 13°. This is because ZnO itself is a polar molecule and has certain hydrophilic properties. After depositing Ag_3_PO_4_, the contact angle is always 0° no matter how many times it is deposited. Since ZnO is a polar molecule as a metal oxide, Ag_3_PO_4_ is a metal inorganic salt and is also a substance having a polar structure. Polar molecules are charged due to internal imbalance, so the two are highly affinitive with polar water due to mutual electrostatic attraction. Therefore the static contact angle is always 0°. The sample is completely hydrophilic so that it is sufficiently in contact with the solution.

**Figure 6:**
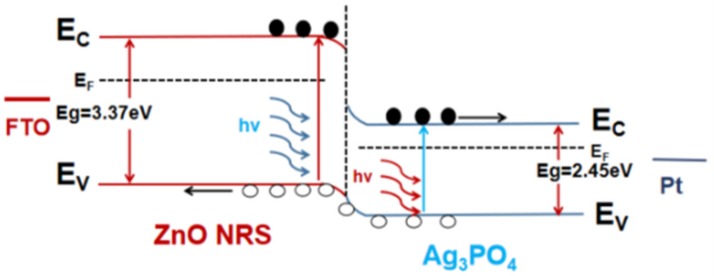
Schematic representation of the ZnO@Ag3PO4 heterojunction.

Figure 6 shows a schematic diagram of the photocurrent generated by the ZnO@Ag_3_PO_4_ heterojunction under illumination. When ZnO nanorods and Ag_3_PO_4_ are in contact with each other, a heterojunction is formed. After the bias is applied, an energy band structure as shown in Figure 6 is formed. When light illuminates two materials, the electrons in the valence band of the two materials will transition to the conduction band. In addition, it will leave holes in the valence band. Under the applied bias voltage, the electrons flow to the conduction band of Ag_3_PO_4_ and flow out through the external Pt electrode. The holes migrate from the Ag_3_PO_4_ valence band to the ZnO valence band and are transported to the external circuit through the FTO conductive glass together with the holes on the ZnO valence band.

**Figure 7:**
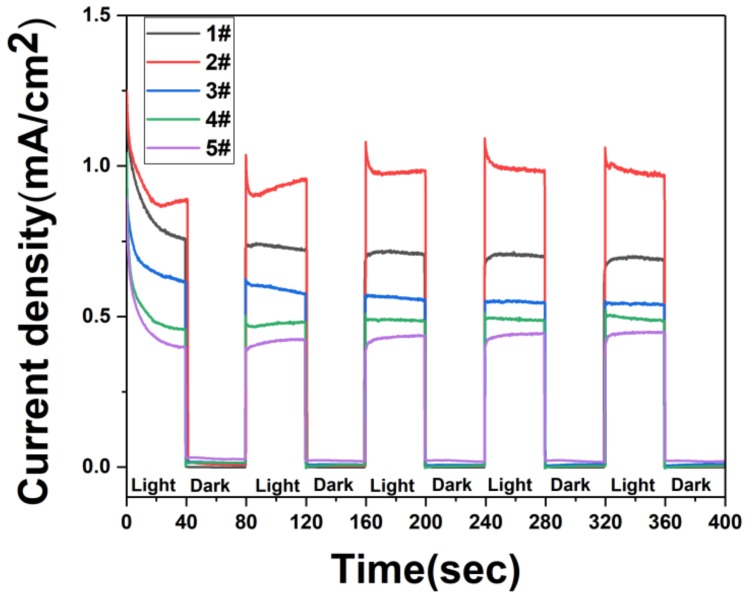
The i-t curve of the sample was prepared in a 0.1 M Na_2_SO_4_ (pH = 7) electrolyte under the action of a constant potential of 0.3 V vs. SCE: (1#) ZnO nanorods. (2#) ZnO nanorods + Ag_3_PO_4_ (1 deposition). (3#) ZnO nanorods + Ag_3_PO_4_ (3 depositions). (4#) ZnO nanorods + Ag_3_PO_4_ (5 depositions). (5#) ZnO nanorods + Ag_3_PO_4_ (7 depositions).

Figure 7 shows the transient photoresponse current under dark/visible light cycling conditions over time with a fixed bias voltage of 0.3 V. Five repeated cycles show that the photocurrent decreases with time during the illumination process and eventually stabilizes, which is related to the stability of the sample. As shown in Figure 7, the current of each sample under illumination conditions is greater than that under dark conditions. Because the electrons on the valence band of Ag_3_PO_4_ and ZnO cannot obtain sufficient energy in the dark condition, they cannot transit into the conduction band, and no photogenerated carrier is generated, and transient photocurrent cannot be formed.

In addition, we also observe that the value of the photocurrent of Ag_3_PO_4_ deposited on the surface of ZnO first increases and then decreases. When Ag_3_PO_4_ is deposited once, the photocurrent value reach 1.3 mA/cm^2^. After a continuous photoresponse test, the photocurrent value is stable at around 1.0 mA/cm^2^. The photocurrent value decrease with the increase of the time of Ag_3_PO_4_ deposited on the surface. When Ag_3_PO_4_ is deposited 7 times, the photocurrent value dropped to about 0.4 mA/cm^2^. When there is only ZnO material, the light illuminates the sample and the electrons receive sufficient energy to transition to the conduction band. Because of the single material, the electron-hole pair recombination efficiency is very high, and the electrons in the conduction band release energy back into the valence band and recombine with the holes in the valence band [63,64,65]. After depositing a small amount of Ag_3_PO_4_ on the surface of ZnO, Ag_3_PO_4_ material can also effectively absorb photons and improve the utilization of light. After the heterojunction is formed between ZnO and Ag_3_PO_4_, the recombination of electron-hole pairs can be effectively suppressed, and the electron-hole pairs migration can be enhanced to promote the enhancement of photocurrent [66,67]. As the time of surface deposition of Ag_3_PO_4_ increases, the increase in the number of surface Ag_3_PO_4_ leads to the increase of surface resistance, which ultimately reduces the photocurrent.

**Figure 8:**
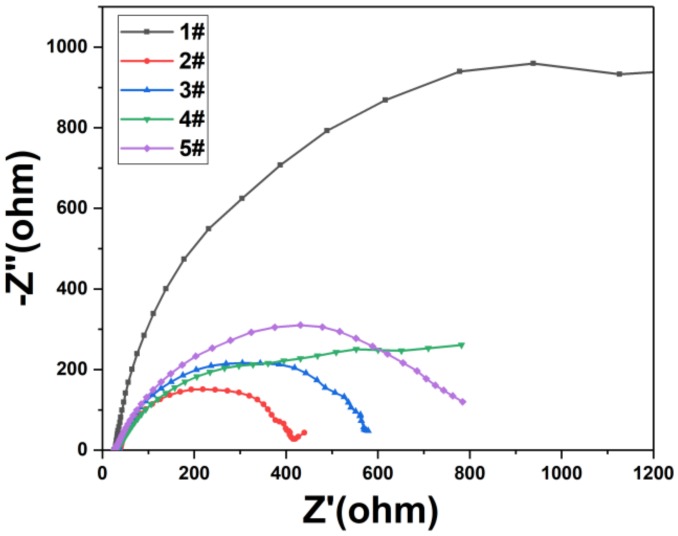
Nyquist diagram of the samples under visible light illumination: (1#) ZnO nanorods. (2#) ZnO nanorods + Ag_3_PO_4_ (1 deposition). (3#) ZnO nanorods + Ag_3_PO_4_ (3 depositions). (4#) ZnO nanorods + Ag_3_PO_4_ (5 depositions). (5#) ZnO nanorods + Ag_3_PO_4_ (7 depositions).

In order to better reveal the principle of photocurrent response, we conducted AC impedance test results as shown in Figure 8. We observe that when only ZnO nanorods are used, the impedance value is very high, and the impedance value is greatly reduced after the deposition of Ag_3_PO_4_ to form a heterojunction. As the time of depositions of Ag_3_PO_4_ increases, the impedance value also increases. Comparing Figure 7 and Figure 8, we observe that the impedance of the samples with only ZnO nanorods is higher than the impedance of the samples with multiple depositions of Ag_3_PO_4_, but the photocurrent value of the samples with only ZnO nanorods is higher than that of the samples with multiple depositions of Ag_3_PO_4_. Because a small amount of Ag_3_PO_4_ is deposited on the surface, the formation of a heterojunction between ZnO and Ag_3_PO_4_ can suppress the recombination of electron-hole pairs and accelerate the separation of electron-hole pairs, so that the photocurrent can be effectively enhanced by illumination. However, as the deposition of Ag_3_PO_4_ on the surface of ZnO nanorods increases, Ag_3_PO_4_ will cover the ZnO nanorods on a large surface, and the holes in the ZnO valence band will remain in the ZnO material. As the number of holes trapped in the ZnO nanorods increases, the photocurrent formed is weakened and the ZnO nanorods are also corroded. For samples with only ZnO nanorods, this is not the case. Therefore, we can obtain the best photoelectric performance of Ag_3_PO_4_ deposited on the surface of ZnO nanorods once.

## 4. Conclusions

In this study, we successfully prepared ZnO@Ag_3_PO_4_ core-shell nanocomposite arrays structure by the hydrothermal method and the stepwise deposition method. The morphology, structure and elements of the material were analyzed by SEM, XRD and EDS. Light reflection indicates that deposition of Ag_3_PO_4_ on the surface of ZnO nanorods can effectively reduce the reflection of light. The deposition of Ag_3_PO_4_ can simultaneously make the surface of the material superhydrophilic. When the ZnO@Ag_3_PO_4_ core-shell nanocomposite arrays structure is used as a photoanode for photoelectric performance testing, through photocurrent response test, we find that depositing a small amount of Ag_3_PO_4_ on the surface of ZnO nanorods can enhance photocurrent. When Ag_3_PO_4_ is deposited once on the surface of ZnO nanorods, the transient photocurrent reached 1.3 mA/cm^2^, and the photocurrent was stabilized at 1.0 mA/cm^2^ after repeated cycles. Through the alternating current (AC) impedance analysis, we find that the ZnO@Ag_3_PO_4_ core-shell nanocomposite arrays have the lowest impedance when Ag_3_PO_4_ is deposited once on the surface of ZnO nanorods. Therefore, the ZnO@Ag3PO4 core-shell nanocomposite arrays structure can be applied to high-speed photoelectric switches, solar cells, sensors and other fields.
